# No self‐serving bias in therapists' evaluations of clients' premature treatment termination: An approximate replication of Murdock et al. (2010)

**DOI:** 10.1002/cpp.2677

**Published:** 2021-11-04

**Authors:** Brechje Dandachi‐FitzGerald, Laura Meijs, Isabelle M. A. J. Moonen, Harald Merckelbach

**Affiliations:** ^1^ Department of Clinical Psychological Science Maastricht University Maastricht The Netherlands

**Keywords:** need for closure, psychotherapy, replication study, self‐confidence, self‐serving bias, therapy dropout

## Abstract

In an often‐cited study, Murdock et al. (2010) found that therapists are more likely to attribute premature treatment termination to client characteristics than to themselves, a finding that the authors interpreted in terms of a self‐serving bias (SSB). We replicated and extended the study of Murdock et al. (2010, study 2). Psychologists and psychotherapists (*N* = 91) read two case vignettes about premature treatment terminations of clients that, in a between‐subjects set‐up, were either described as own clients or other therapists' clients. Next, participants used three attribution subscales (blaming therapist, client and situation) to evaluate potential causes for the premature terminations. This way, we tested whether participants would manifest SSB. We also investigated whether therapists' scores on self‐confidence and need for closure were linked to SSB tendencies. Unlike Murdock et al. (2010), we found no overall SSB. However, a stronger need for closure was related to more SSB tendencies (i.e., less endorsement of ‘blame therapist’ attributions) in the own‐client condition (*r* = −.35, *p* < .05, *r* ^2^ = .12), but not in the other‐therapist's‐client condition (*r* = .17, *p* = .27). Our results suggest that SSB is not a ubiquitous phenomenon when therapists evaluate premature termination problems and that their willingness to attend to their own role depends to some extent on their need for closure.

**Key Practitioner Message**

- In contrast to Murdock et al. (2010), we did not find a self‐serving bias (SSB) in how therapists evaluate clients' premature treatment termination.
- Our findings suggest that SSB is not a ubiquitous phenomenon when therapists evaluate therapy dropout.
- SSB might be related to specific therapists' characteristics. In particular, therapists with a strong need for closure might have more difficulty in acknowledging their own role in premature termination.
- Future studies should further explore the role of SSB and therapist characteristics such as need for closure in dealing with negative treatment outcomes.
- Replication studies are important to determine the robustness, generalizability and boundaries of claimed effects.

## 1 INTRODUCTION

Approximately 20% of clients in psychological treatments unilaterally decide to discontinue therapy (Cooper & Conklin, 2015; Fernandez et al., 2015; Linardon et al., 2019; Swift & Greenberg, 2012), an outcome referred to as *premature termination* or *therapy dropout* (Hatchett & Park, 2003).

Therapists and clients do not always share the same understanding of the reasons for premature therapy termination. For example, Westmacott et al. (2010) asked therapist–client dyads of therapies that had ended unilaterally by the client (*n* = 31) versus by mutual agreement (*n* = 52) to rate 10 possible reasons for termination and their importance in the decision to end therapy. The perspectives of clients and therapists differed more in unilateral termination than in mutual agreement cases. In unilateral termination cases, the largest discrepancies were evident for four items related to dissatisfaction with the therapy (i.e., ‘Felt therapy was making things worse, so stopped’, ‘Felt therapy was going nowhere so ended therapy’, ‘Weren't confident in therapist's ability to help’ and ‘Therapy didn't fit with my idea about what would be helpful’). Clients rated these items as more important for ending therapy than did their therapists. Werbart et al. (2019) interviewed three therapists and their clients who had been involved in three successful and three unsuccessful treatments as determined by change scores on a symptom list. Whereas therapists' and clients' views were aligned for successful therapies, their views starkly diverged for unsuccessful therapies. In unsuccessful therapies, clients experienced mistrust, felt misunderstood and indicated that there was no match. Therapists, on the other hand, indicated that the client did not want to talk about certain traumatic experiences or that more time was needed because the client resisted. Thus, although therapists were able to recognize difficulties in the therapeutic relationship, they mainly attributed it to the client's pathology and seemed to disregard their own role.

Arguably, a discrepancy between clients' and therapists' attributions for premature therapy termination is undesirable. Therapists might miss the opportunity to intervene and to reduce the risk of therapy failure when they overlook clients' dissatisfaction with the treatment (Roos & Werbart, 2013; Westmacott & Hunsley, 2017). More generally, by focusing on negative treatment experiences, therapists may improve their expertise (Ericsson, 2009). Why, then, is it difficult for therapists to recognize their own role in premature termination? One explanation might be that therapists, when evaluating premature therapy termination, suffer from a bias known as self‐serving bias (SSB).

### 1.1 SSB and premature termination

SSB can be defined as the proclivity to attribute positive personal outcomes to oneself and negative personal outcomes to situational or external causes so as to protect one's self‐concept (Campbell & Sedikides, 1999; Heider, 1958; Taylor & Brown, 1988). SSB is considered to be an adaptive heuristic of judgemental evaluations (Mezulis et al., 2004; Sedikides & Alicke, 2019; Shepperd et al., 2008). Still, in the context of therapeutic relationships, self‐serving attributions on the part of the therapist are likely to hinder acknowledgement and remedy of problems, which consequently may encourage clients to prematurely terminate the relationship due to dissatisfaction with services (Manfred‐Gilham et al., 2002).

To the best of our knowledge, a study of Murdock et al. (2010) is the only one that experimentally investigated how psychotherapists attribute causes to premature therapy ending. On the basis of their findings, these researchers contend that therapists do exhibit SSB when they are asked to rate causes that could have contributed to the premature therapy termination. More precisely, Murdock et al. (2010) noted that therapists put more blame for premature termination on the client when evaluating a vignette involving a person presented as their own client and they put more blame on the therapist when evaluating a vignette involving another therapist's client.

### 1.2 Therapists' self‐confidence and need for closure

There might exist individual differences between therapists in their tendency to display SSB when confronted with therapy dropout. Specifically, therapists' confidence in their therapy skills and their need for closure might be relevant candidates.

As to confidence, Walfish et al. (2012) found that 25% of the mental health professionals they surveyed viewed their therapeutic skills to be at the top 10 per cent when compared with those of their colleagues. Also, none of the surveyed professionals scored their therapeutic skills as below average. Arguably, premature termination might be especially a threat to therapists who place much confidence and trust in their own therapy skills, and therefore, this group might engage more in self‐serving attributions to protect their self‐esteem than do therapists who are humble about their skills (Campbell & Sedikides, 1999). In line with this, Murdock et al. (2010) found that relative to low‐confidence participants, participants with high confidence in their therapeutic skills were less likely to attribute premature termination to therapist factors in the own‐client condition.

Need for closure (NFC) refers to a need for certainty, a desire for predictability, a preference for structure and order, and to feelings of discomfort when things seem vague (McKay et al., 2006; Webster & Kruglanski, 1994). Although NFC can increase under certain circumstances, such as time pressure or tiredness, people differ in their chronic level of ‘dispositional closure’ (McKay et al., 2006; Roets & Van Hiel, 2011; Webster & Kruglanski, 1994). People high in NFC readily focus on the favourable aspects of the self and reinterpret deviant information, allowing them to persist in their initial interpretations. People with low NFC have the tendency to look critically at behaviours of others and themselves, thus moderating their self‐enhancing beliefs (Ford & Kruglanski, 1995; Taris, 2000). Therefore, one would expect that high NFC therapists exhibit a stronger SSB (i.e., to focus more readily on client and situational reasons) when confronted with premature ending than low NFC therapists.

### 1.3 The current study

The Murdock et al. (2010) paper is an influential study that is often cited as though it were a secured part of the corpus of knowledge on therapist–patient interactions. For example, referring to the Murdock et al. study, Piselli et al. (2011, p. 400) opined that, ‘While therapists may assume personal responsibility for the unexplained outcome, they are more likely to attribute premature termination to client factors.’ We wanted to test how robust the SSB effect is that Murdock et al. (2010) documented. With this in mind, we conducted an approximate replication of their study (American Psychological Association, 2020). Thus, by employing a similar experimental set‐up and comparable case vignettes, we examined whether Dutch psychotherapists and psychologists exhibit SSB when asked for reasons for clients' premature termination. We also wanted to find out whether therapists' confidence in their therapeutic skills and their NFC scores are positively and significantly correlated with their SSB.

## 2 METHOD

### 2.1 Replication details

We tried to adhere as closely as possible to the original set‐up. After initial contact with the first author of the original study, we did not receive the original material and therefore assumed it is no longer available. Consequently, we had to resort to the information in the published paper to construct the material (e.g., the case vignettes) and were not able to carry out an exact (literal) replication. Our replication study differed in four other ways from the original study. First, we used an online platform instead of paper‐and‐pencil questionnaires administered via regular postal services. Second, we added experimental checks to screen for inattentive responding. Third, in the statistical analysis, we entered case vignette as a separate within‐subject variable whereas Murdock et al. (2010) averaged scores for the two case vignettes and entered that collapsed variable in their 2 (condition) × 3 (attribution scales) repeated measures analysis. Finally, we explored whether therapists' self‐confidence and NFC are related to SSB tendencies.

### 2.2 Participants

We recruited Dutch speaking psychotherapists, health care psychologists (i.e., certified psychologists with 2‐year postgraduate training), clinical psychologists and clinical neuropsychologists (i.e., 6‐year postgraduate training), including those in training, who provided any form of psychological treatment in their current position/profession. We focused on these groups because we assumed that they had substantial training and several years of work experience. Recruitment took place within the network of the authors and via advertisements on social media (e.g., LinkedIn), the website of the Dutch association for certified psychologists and the Dutch association for psychotherapy. An a priori power analysis using G*Power (Faul et al., 2007) with an alpha of .05, power set at .80 and assuming a small effect size of 0.14 (as per Murdock et al., 2010) resulted in a required total sample size of *N* = 72. Anticipating exclusion of some participants due to failure to comply with the experimental instructions (e.g., Oppenheimer et al., 2009), we aimed at recruiting 80 participants.

In total, 139 participants started the questionnaire of whom 110 completed all items. Non‐completers (*n* = 29) did not differ from completers with regard to age, *t*(137) = .76, *p* = .45, and gender (Fisher's exact *p* = .49). Incomplete questionnaires were excluded. Four participants indicated that they did not work as a psychologist and their data were also removed. Finally, 15 questionnaires of the remaining 106 eligible participants (14.2%) were excluded from data analysis because of inattentive responding (i.e., failing the experimental check items), leaving 91 participants in the final sample.

### 2.3 Research design

The study relied on a 2 (between‐subjects: own client vs. another therapist's client) × 2 (within‐subjects: case vignette 1 vs. case vignette 2) design.

### 2.4 Measures

#### 2.4.1 Demographic questionnaire

The following demographic and therapist data were obtained: age, gender, professional title, main theoretical orientation (1 = based on learning theory principles; 2 = psychoanalytical/psychodynamic, 3 = client centred), current work setting, number of work hours, estimated percentage of work hours spent on providing psychological treatment and estimated percentage of clients in their practice who terminate treatment unilaterally and prematurely.

#### 2.4.2 ‘Confidence in own therapy skills’ measurement

Three items taken from Walfish et al. (2012) assessed therapists' confidence in their clinical skills: (1) ‘How self‐confident are you in your psychotherapy skills (0–100%, with 25% = below average, 50% = average, and 75% = above average)?’; (2) ‘Compared with other mental health professionals within your field (with similar credentials), how would you rate your overall clinical skills and performance in terms of a percentile (0–100%, with 25% = below average, 50% = average, and 75% = above average)?’; and (3) ‘What percentage (0–100%) of “clients in general” (in another‐therapist's‐client condition) versus your clients (in own‐client condition) get better (i.e., experience significant symptom reduction) during treatment? What percentage stays the same? What percentage gets worse?’. The average score on the first two questions was used as a proxy measure of self‐confidence of therapists.

#### 2.4.3 Brief need for closure scale (brief NFC)

The Dutch version of the brief NFC scale consists of 15 statements of which participants have to indicate how much they agree with each on a 6‐point Likert scale (1 = *strongly disagree*; 6 = *strongly agree*). Sample items are as follows: ‘When I have made a decision, I feel relieved’ and ‘I dislike unpredictable situations’. The psychometric properties of the brief NFC scale are comparable to the original 42‐item NFC scale (Webster & Kruglanski, 1994), with almost a similar internal consistency (Cronbach's alpha .90 vs. .87) (Roets & Van Hiel, 2011). Cronbach's alpha in the current study was .80.

#### 2.4.4 Case vignettes

We designed two Dutch case vignettes, based on the descriptions in Murdock et al. (2010). Vignettes were about two female clients who had similar psychopathology and made significant therapy improvement, but identified remaining emotional issues. Both terminated treatment prematurely and unilaterally without informing the therapist about their motives to do so. In order to examine SSB, the cause for premature treatment termination had to remain ambiguous. Thus, following Murdock et al. (2010), the therapeutic relation was presented in the vignettes as unproblematic so as to avoid that participants would list it as the obvious cause of premature termination.

One client, Marij, sought professional help because of grief issues after her partner died in a car accident. The second client, Kirsten, was characterized as lonely, stressed and unhappy because of geographical and study/career changes. Both vignettes ended with a report that before last week's session, the client called and terminated therapy without an explanation.

#### 2.4.5 Causal Attributions Questionnaire

The Causal Attributions Questionnaire (CAQ) was taken from Murdock et al. (2010, study 1) and consists of 11 categories of potential causes of premature therapy termination. Each causal category is assessed on a 7‐point Likert‐type scale ranging from 1 = *very unlikely* to 7 = *very likely*. Furthermore, an ‘other’ item was added that allowed participants to suggest and weight an additional cause not covered by those listed. In the current study, we focused on the three attribution scales of the CAQ as per Murdock et al. (2010): (1) the therapist; (2) the client; and (3) the situation. The first scale (*α* = .79) consists of categories that consider the therapist to be the primary cause for premature termination (e.g., the therapist's inability or incompetence and the client's perception of the therapist). The second scale (*α* = .76) is composed of categories referring to the client as a cause for premature termination (e.g., anxiety, discomfort or resistance of the client, level of motivation and expectations). The third scale (*α* = .76) is composed of categories related to external situations (e.g., financial matters, insurance issues and reactions from relatives). One item of the CAQ was not included in the attribution scales. This item pertains to clients ending therapy because they feel better. Data obtained with this item were analysed separately.

#### 2.4.6 Experimental checks

With control questions at various places, we checked whether respondents had read the vignettes and responded attentively. Thus, after reading the case vignettes, participants were asked to answer two multiple choice questions about the content (e.g., what sport does Kirsten practice?). Two items were added to the brief NFC scale: ‘When I am reading this well, I fill in strongly agree’ and ‘I have never spoken with anyone who wears glasses’. Three post‐experimental questions queried whether participants had read all questions carefully and answered them accurate and truthfully, what participants thought the aim of the study was and whether they were familiar with papers about therapists' self‐confidence.

### 2.5 Procedure

The study was conducted using the online platform Qualtrics. Therapists were recruited through advertisement that invited them to participate and to opt in by activating a link directing them to a consent page and an introductory text in which the two conditions were not mentioned. The text stated that reading the two case vignettes and answering the questions would take around 20 to 30 min and that there was no compensation for participation. It was also explicitly made clear that data were collected anonymously.

After signing the informed consent form, participants were randomly assigned to either the own‐client or the another‐therapist's‐client condition. In the own‐client condition, the client was referred to as ‘your client, Marij (or Kirsten)’ and the rest of the vignette used the pronouns ‘you’ or ‘your’ when appropriate. In the another‐therapist's‐client condition, references were always to ‘the therapist’ and ‘the client’.

First, participants received the demographical questionnaire, the ‘confidence in therapy skills’ items and the brief NFC scale. Next, they read the first case vignette (Marij) and filled out the CAQ, followed by the second case vignette (Kirsten) and the CAQ. In case the categories of the CAQ were unclear, participants were given the opportunity to open an extra document that explained each category with several examples taken from Murdock et al. (2010, study 1). Finally, the post‐experimental checks were administered. Upon completion, participants were fully debriefed about the aim, experimental manipulation and hypothesis of the study. Ethical approval was obtained from the standing Ethical Review Committee of the Faculty of Psychology and Neuroscience at Maastricht University (ERCPN‐221_58_03_2020).

### 2.6 Data analysis

Preliminary analyses were conducted to determine whether background characteristics (i.e., gender, age, profession, work setting, work hours, theoretical orientation, therapists' self‐confidence in therapeutic skills and need for closure) were similar for the two conditions. Next, we conducted a 2 (conditions) × 2 (case vignettes) × 3 (attribution scales: therapist, client and situation) analysis of variance (ANOVA) with repeated measures on the last two factors. To test whether therapists exhibited SSB, we were specifically interested in whether participants in the own‐client condition would endorse more often situational or client‐related attributions than participants in the another‐therapist's‐client condition, a constellation that would be flagged by a significant condition by attribution scales interaction.

Finally, we explored with Pearson correlations the links therapists' self‐confidence, their need for closure and the attribution scales (therapist, client and situation) across the two conditions separately. All statistical analyses were performed using SPSS Version 25.

## 3 RESULTS

### 3.1 Preliminary analyses

Table 1 shows background characteristics of participants. There were no differences between conditions with regard to any of the background variables (all *p*'s ≥ .23). For the total sample, a higher level of self‐confidence in therapeutic skills was related to less need for closure (*r* = −.26, *p* < .05, *r* ^2^ = .07). Furthermore, age was associated with self‐confidence (*r* = .28, *p* < .01, *r* ^2^ = .08). The relationship between age and need for closure attained borderline significance (*r* = −.20, *p* = .05). Therapists' self‐confidence in therapeutic skills and need for closure were not related to other baseline characteristics, that is, number of work hours, estimated percentage of work hours spent on providing psychological treatment and estimated percentage of clients in their practice who prematurely terminate treatment (all *p*'s ≥ .22).

**TABLE 1:** Sample characteristics of participants per condition

Characteristic	Total sample (*N* = 91)				Own‐client condition (*n* = 47)				Another‐therapist's‐client condition (*n* = 44)				*p*
	*N*	%	M	SD	*n*	%	M	SD	*n*	%	M	SD	
Gender^a^													.23
Women	81	89.0			44	93.6			37	84.1			
Men	9	9.9			3	6.4			6	13.6			
Non‐binary	1	1.1			0	0.0			1	2.3			
Age (years)^b^			37.7	10.3			37.3	9.6			38.1	11.0	.69
Profession^a^													.75
Psychotherapist	9	9.9			5	10.6			4	9.1			
Psychotherapist trainee	6	6.6			4	8.5			2	4.5			
Health care psychologist^c^	36	39.6			20	42.6			16	36.4			
Health care psychologist trainee	25	27.5			11	23.4			14	31.8			
Clinical psychologist	9	9.9			3	6.4			6	13.6			
Clinical psychologist trainee	3	3.3			2	4.3			1	2.3			
Clinical neuropsychologist	1	1.1			1	2.1			0	0.0			
Clinical neuropsychologist trainee	1	1.1			0	0.0			1	2.3			
Other	1	1.1			1	2.1			1	0.0			
Work setting^a^ ^,^ ^d^													.62
Independent practice	15	16.5			8	17.0			7	15.9			
Mental health care centre	71	78.0			37	78.7			34	77.3			
Hospital	10	11.0			6	12.8			4	9.1			
Other	7	7.7			3	6.4			4	9.1			
Main theoretical orientation^a^ ^,^ ^d^													.56
Based on learning principles (e.g., CBT, EMDR, and schema therapy)	81	89.0			42	89.4			39	88.6			
Client centred (e.g., EFT)	20	22.0			12	25.5			8	18.2			
Psychodynamic/psychoanalytical (e.g., MBT)	22	24.2			13	27.7			9	20.5			
Other	6	6.6			3	6.4			3	6.8			
Working hours^b^			30	6.2			30.3	5.3			29.6	7.0	.60
% work providing treatment^b^			56.4	20.5			57.3	18.8			55.3	22.3	.65
% premature termination^b^			7.4	6.1			7.2	5.9			7.5	6.3	.82
Therapists' self‐confidence^b^			63.5	11.56			63.6	10.7			63.4	12.5	.92
Therapists' need for closure^b^			3.4	.53			3.4	0.6			3.5	0.5	.46
Therapists' estimation (%) of therapy result													
Improvement^b^			68.9	11.0			68.1	11.6			67.7	10.5	.87
Unchanged^b^			24.1	9.0			24.1	9.7			24.0	8.3	.92
Deterioration^b^			8.0	5.0			7.8	4.5			8.3	5.6	.59
Attribution scales													
Therapist							4.5	0.9			4.3	0.8	
Client							4.6	0.7			4.5	0.8	
Situation							3.7	0.8			3.8	0.8	
Item ‘client felt better’							4.9	1.1			4.9	1.0	.91

*Note*: For profession, the analysis was performed for the main categories: (1) psychotherapist (trainee); (2) health care psychologist (trainee); and (3) clinical psychologist/clinical neuropsychologist (trainee).

Abbreviations: CAQ, Causal Attribution Questionnaire; CBT, cognitive behavioural therapy; EFT, emotion‐focused therapy; MBT, mentalization‐based therapy.

^a^ *χ* ^2^ analysis.

^b^ Independent samples *t*‐test.

^c^ Certified psychologists with 2‐year postgraduate training.

^d^ Multiple answers could be given. Therefore, the percentages do not add up to 100.

### 3.2 Self‐serving bias

A 2 (condition) × 2 (case vignettes) × 3 (attribution scales) ANOVA with repeated measures on the last two factors indicated that the critical interaction of condition with attribution scales failed to reach significance, *F*(2, 88) = .75, *p* = .48, meaning that there was no SSB.^1^ The main effect of condition remained non‐significant, *F*(1, 89) = .39, *p* = .54: On the whole, participants evaluated own‐client and another‐therapist's‐client version of the vignette similarly. There was a main effect of case vignettes, *F*(1, 89) = 8.35, *p* < .01, partial *η* ^2^ = .09, and of attribution scales, *F*(2, 88) = 28.21, *p* < .01, partial *η* ^2^ = .24. The three‐way interaction of condition, case vignette and attribution scales was not significant, *F*(2, 88) = .84, *p* = .44. Also, there was no significant two‐way interaction between case vignette and condition, *F*(1, 89) = .47, *p* = .49. There was, however, a significant two‐way interaction between case vignettes and attribution scales, *F*(2, 88) = 6.1, *p* < .01, partial *η* ^2^ = .12, indicating that attributions for premature treatment termination differed between the two case vignettes. To locate the source of this interaction, we carried out separate ANOVA's. As can be seen in the supporting information and Figure S1, for vignette 1, participants endorsed client and therapist factors similarly often, whereas for vignette 2, client factors were preferred over therapist factors. For both vignettes, situational factors were less frequently endorsed than either therapist or client factors.

### 3.3 Therapists' characteristics and SSB

We collapsed attribution data across case vignettes and correlated these average values to the therapists' level of self‐confidence in their therapeutic skills and their need for closure for both conditions, separately. Table 2 shows the Pearson‐product moment correlations. As can be seen, there was a negative association between therapist's level of need for closure and the attribution of the premature treatment termination to therapist's factors in the own‐client condition (*r* ^2^ = .12). This relation was absent in the another‐therapist's‐client condition. There was no relationship between self‐confidence in therapeutic skills and attributions for premature termination.

**TABLE 2:** Intercorrelations for therapist's characteristics and attribution scales disaggregated by condition

Variable	1	2	3	4	5
1. Need for closure		−.23	.17	.06	−.24
2. Self‐confidence in therapeutic skills	−.30^*^	‐	−.02	.10	.04
3. Therapist‐related attributions	−.35^*^	−.03	‐	.24	.06
4. Client‐related attributions	.05	−.13	.32^*^	‐	.22
5. Situation‐related attributions	.15	.19	−.01	.04	‐

*Note*: The results for the own‐client condition sample (*n* = 47) are shown below the diagonal. The results for the another‐therapist's‐client condition (*n* = 44) are shown above the diagonal.

^*^ *p* < .05.

## 4 DISCUSSION

The present study aimed to replicate and extend the Murdock et al. (2010) study in a sample of Dutch psychologists and psychotherapists. With two hypothetical case vignettes that varied only in the relationship of the therapist to the client (i.e., own client vs. another therapist's client), we examined whether therapists' causal attributions exhibited an SSB. In addition, we examined two potential correlates of SSB, namely, therapists' level of self‐confidence in their therapeutic skills and therapists' level of need for closure. Our findings can be summarized as follows. First, in contrast to Murdock et al. (2010), we did not find evidence for a general SSB in therapists' attributions. That is, when evaluating reasons for premature termination, participants in the own‐client condition did not more often endorse external causes (i.e., client and/or situational factors) compared with participants in the another‐therapist's‐client condition. Second, irrespective of condition, participants attributed more causal weight to factors related to the client and therapist than to the situation.

Our failure to obtain an SSB is remarkable given that this bias is such a widely acknowledged phenomenon that is prevalent in many samples and across a wide array of situations (Mezulis et al., 2004). Why were we unable to replicate the SSB observed by Murdock et al. (2010)? For one thing, the current study involved mainly women, which is relevant because the extant literature suggests that men are more sensitive to SSB than women (e.g., Mezulis et al., 2004; Shepperd et al., 2008). Because Murdock et al. (2010) had similar numbers of men and women in their sample and found a larger SSB in men (*η* ^2^ = .17) than in women (*η* ^2^ = .06), this can partially explain the inconsistent findings.

Second, our study was an approximate rather than a precise replication attempt, due to the fact that we had to construct our own case vignettes. These vignettes were based on the description provided in Murdock et al. (2010). Still, our case vignettes might, for unknown reasons, elicit fewer SSB tendencies than those of Murdock et al. (2010).

Relatedly and third, Campbell and Sedikides (1999) demonstrated that SSB can be amplified by material that increases feelings of self‐threat. When individuals experienced little self‐threat, no self‐serving attributions were observed (Campbell & Sedikides, 1999). Therefore, a possible explanation for our null findings might be that participants experienced minimal self‐threat when the clients in the fictional case vignettes terminated prematurely. Perhaps, therapists were convinced that the clients in the fictional case vignettes, Marij and Kirsten, stopped treatment because they felt better. If so, the unilateral termination might not be interpreted as a failure. Indeed, in the current study, 64.8% of the participants rated the cause ‘client felt better’ as somewhat to very likely contributing to the premature termination of the clients in the fictional case vignettes. Of note, the average rating for ‘the client felt better’ did not differ between the two conditions (own client vs. another therapist's client).

Fourth, 14% of the participants in our sample responded inattentively (i.e., failed to read or attend to item content). Inattentive responding adds error variance to the data and may obscure existing relationships or produce spurious relationships in hypothesis testing. Inattentive responding is common in experimental and survey studies and its impact on data quality has been well documented (e.g., Maniaci & Rogge, 2014; Oppenheimer et al., 2009). The study of Murdock et al. (2010) did not include experimental check items to control for inattentiveness. Thus, it is unknown to what extent their findings were impacted by error variance introduced by careless responders.

Fifth and last, an obvious difference between Murdock et al. (2010) and our study is that the first relied on a sample of US therapists whereas our sample involved Dutch psychotherapists. We are not aware of any prominent difference in the training of therapists between the two countries that could be relevant for our study. Still, there might be cultural differences factors operating in the training and practice of psychotherapists in both countries that may explain the divergent findings. There were, for example, differences between the work settings of the therapists in both samples. In the study of Murdock et al. (2010), most therapists worked in an independent setting (57.5%), whereas in our sample, most therapists worked in a mental health care institute (78.0%). Also, differences in the mental health care system (e.g., health coverage, financial barriers etc.) exist between the two countries (Sareen et al., 2007). It might well be the case that these differences in setting enhance or suppress SSB. Obviously, this an issue that requires further exploration.

We did not find evidence for a robust and overall SSB in how therapists evaluate case vignettes that might signal treatment failure (i.e., premature termination). However, our correlational data provide some interesting hints to a connection between SSB and therapists' need for closure. A higher need for closure was related to attributing less blame to therapist factors in the own‐client condition. This relation was absent in the another‐therapist's‐client condition. The suggestion that SSB is linked to therapist's characteristics fits well with the larger research database on individual differences between therapists—so‐called *therapist effects*—on psychotherapy outcome (Johns et al., 2019; Saxon et al., 2017; Zimmermann et al., 2017). Research on positive therapist effects found most evidence for alliance bond capacity and limited evidence for therapists' facultative interpersonal skills, professional self‐doubt and engagement in deliberate practice (see, for an overview, Hill & Castonguay, 2017). Need for closure might be on the opposite side of these positive characteristics and may increase the risk for suboptimal treatment outcomes. That is, clinicians who have a need for clear answers might be less inclined to engage in professional self‐doubt, to reflect upon the therapy process and to critically look at their own role in therapy outcome. Clearly, this issue warrants further research.

### 4.1 Limitations

Several limitations of the current study warrant comment. First, we constructed case vignettes based on the description in Murdock et al. (2010) and did not check for the number of possible client, therapist and situational factors in each vignette. In case vignette 2 (Kirsten), there were more factors that alluded to situational barriers as opposed to case vignette 1 (Marij). Future research should, therefore, use ambiguous client case vignettes in which client, therapist and situational factors are incorporated in a more balanced way and pre‐experimentally evaluated. Also, to test for order effects, case vignettes should be presented in a counterbalanced manner.

As discussed, the vignettes may not have been perceived as threatening to the self‐esteem of the therapists. Using videos to present vignettes could possibly deepen our understanding of therapists' reactions and termination attributions. The another‐therapist's‐client condition could then be depicted by having therapists observe a video of another therapist with his/her client. In addition, premature contentment of the client's side could also lead to premature termination and if known to the therapist, not negatively affect their self‐esteem. Thus, future researchers might want to focus on the relation between specific reasons for premature termination and experienced self‐threat.

Relatedly, even though we added an extra item compared with Murdock et al. (2010), our measure of therapists' self‐confidence was rather coarse.

The current study heavily relied on female participants. A meaningful analysis of sex differences, as was done in the Murdock et al. (2010) study, was impossible in our study given the low number of men (*n* = 9). In the Netherlands, about 14% to 32% (depending on registration) of the general therapist population is male (Capaciteitsorgaan, 2018). In the United States, about 45% of the practicing members of the American Psychological Association (APA) was male in 2006 (Murdock et al., 2010). Therefore, the gender distributions in both study samples quite accurately reflect the gender ratio in clinical practice at the time of the data collection.

Another limitation is that unlike Murdock et al. (2010), we were unable to compare theoretical orientations because participants did report multiple approaches. A solution might be to force participants to choose their preferred theoretical approach in the psychological treatments they provide (instead of allowing multiple answers). However, the obtained categories might be quit artificial and not accurately reflect clinical practice. Also, consistent with the literature on therapist effects (e.g., Hill & Castonguay, 2017), exploring individual therapists' characteristics seems more fruitful than focusing on theoretical orientations.

Apart from the above listed limitations, there is a more fundamental issue that is not covered by research of the sort described in this paper and Murdock et al. (2010). Specifically, attribution and evaluations of therapy success of the people who are at the heart of the therapeutic process, that is, clients and patients, are not systematically taken into account. Thus, future studies might want to develop an experimental approach that includes both therapists' and clients' attributions of premature treatment ending and their interactions (see also Werbart et al., 2019). Finally, both the original study and our approximate replication study used two vignettes illustrating a negative treatment outcome. Arguably, using case vignettes that differ in outcome (e.g., improvement vs. worsening of symptoms during treatment) could be a better way to examine SSB tendencies in therapists' and clients' causal attributions of treatment outcome.

## 5 CONCLUSIONS

The present study did not find evidence for a robust SSB in therapists' evaluation of causes for premature treatment termination. However, our correlational data are suggestive of a link between therapist's characteristics and SSB. A stronger need for closure was related to less attribution to therapist‐related causes for premature termination in the own‐client condition (i.e., less attribution to oneself). This relation was absent in the another‐therapist's‐client condition. Our results suggest that therapists with a strong need for closure might experience more difficulties in attending to their own role for premature termination.

Our point is not that SSB is absent in experimental designs. One important consideration here is that Murdock et al.'s (2010) study and, by implication, our replication attempt relied on only two case vignettes. It might well be the case that with exposure to a richer and more variable range of cases, therapists do exhibit SSB. However, given our results, we do think that for now, we can be confident in our conclusion that SSB is not the robust and easy to elicit bias that many think it is on the basis of the Murdock et al. study. Future studies should further explore the role of SSB and therapist characteristics such as need for closure in dealing with negative treatment outcomes. To further develop professional expertise, there is much to be learned from therapeutic failures.

## CONFLICT OF INTEREST

We have no known conflict of interest to disclose.

## Supporting information


**Figure S1.** Attributions for Client Premature Treatment Termination as a Function of Condition

**Data S1.** Supporting information

## Data Availability

This study was preregistered (osf.io/yn6bh), and all the materials can be accessed on the Open Science Framework: osf.io/yn6bh/files. The data underlying this study have been uploaded to the Maastricht University Dataverse and are accessible using the following link: https://doi.org/10.34894/8QNWZC.

## ENDNOTE

1 As an additional analysis, we performed a 2 (condition) × 2 (case vignette) ANOVA on the ratio therapist/client attributions. Basically, this yielded a similar pattern of findings: The critical main effect of condition was non‐significant, *F*(1, 89) = .49, *p* = .48. Also, the interaction between case vignette and condition failed to reach significance, *F*(1, 89) = .09, *p* = .32. There was a main effect of case vignette, *F*(1, 89) = 4.1, *p* < .05, partial *η* ^2^ = .04.
