# Supplementary material for: No self‐serving bias in therapists' evaluations of clients' premature treatment termination: An approximate replication of Murdock et al. (2010)
Source: Clin Psychol Psychother. 2021 Nov 4;29(3):972–81. doi: 10.1002/cpp.2677 (PMC9298110; doi:10.1002/cpp.2677)

**Supplemental figure**

*Attributions for Client Premature Treatment Termination as a Function of Condition*


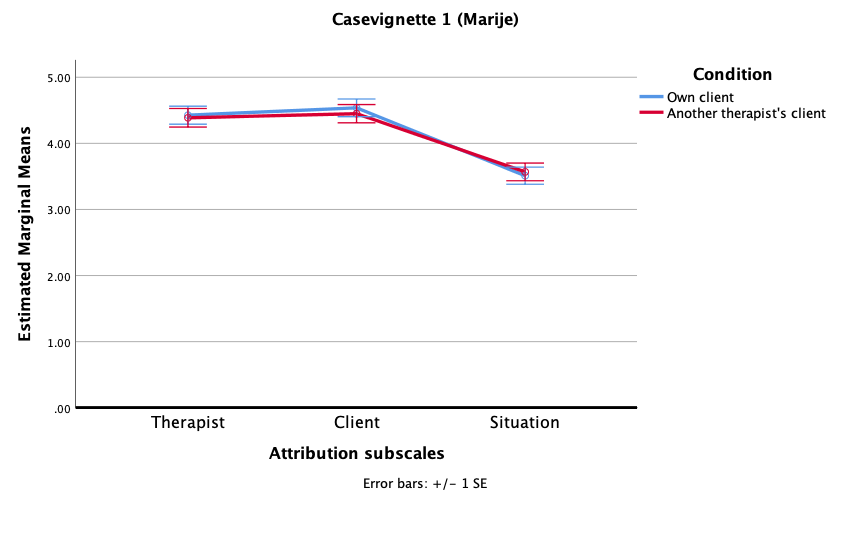


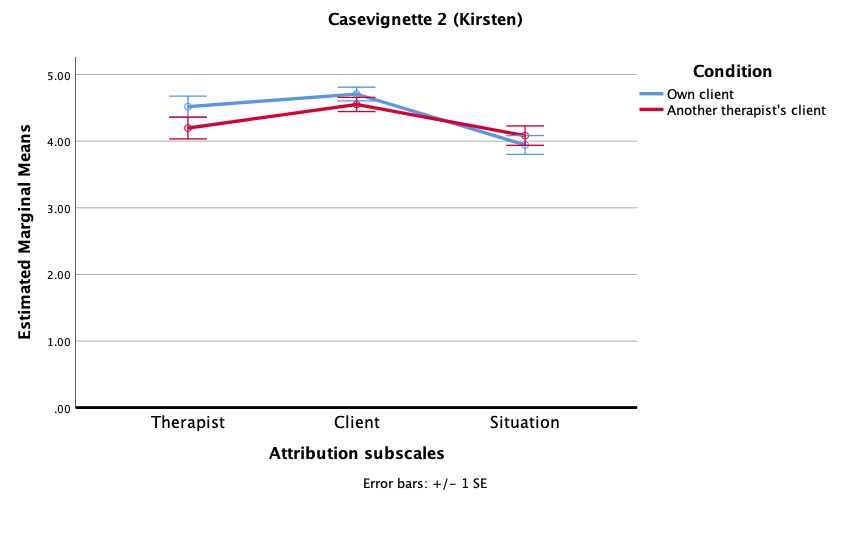

Supplement: Supplementary file 1 — Figure S1. Attributions for Client Premature Treatment Termination as a Function of Condition [file CPP-29-972-s001.docx]
