# Supplementary material for: No self‐serving bias in therapists' evaluations of clients' premature treatment termination: An approximate replication of Murdock et al. (2010)
Source: Clin Psychol Psychother. 2021 Nov 4;29(3):972–81. doi: 10.1002/cpp.2677 (PMC9298110; doi:10.1002/cpp.2677)
Supplement: Supplementary file 2 — Data S1. Supporting information [file CPP-29-972-s002.docx]

**Supplemental file**

*Repeated Measures ANOVA for Casevignette 1 and Casevignette 2.*

For case 1, a 2 (condition) x 3 (attribution subscales) repeated measures ANOVA data did not reveal a significant main effect of condition*, F*(1, 89) = .04, *p* = .85. Neither was there a significant interaction between condition and attribution scales, *F*(2, 88) = .17, *p* = .84. There was, however, a significant main effect of attribution: *F*(2, 88) = 31.02, *p* < .001, partial *η2* = .41. Follow-up *t*-tests indicated that situational factors were less often endorsed than either therapist factors [*t* (90) = -6.20, *p* < .01, Cohen’s *d* = 0.98] or client factors [*t* (90) = -7.78, *p* < .01, *d* = 0.49]. Participants did not prefer client factors over therapist factors, *t* (90) = .76, *p* = .45, *d* = -0.10.

For case 2, there was no main effect of client condition, *F*(1, 89) = .74, *p* = .39. Nor was there a signiﬁcant interaction effect between condition and attribution scales, *F*(2,89) = 1.71, *p* = .19. But, again, a significant main effect of attribution, *F*(2,89) = 12.0, *p* < .001, partial *η2* = .12, emerged. Follow-up *t*-tests indicated that situational factors were less often endorsed than therapist [*t* (90) = -2.41, *p* <.05, Cohen’s *d* = 0.36] or client factors [*t* (90) = -5.14, *p* < .01, *d* = 0.64]. Unlike the pattern evident for vignette 1, participants endorsed more often client than therapist factors in case 2, *t* (90) = 2.45, *p* <.05, *d* = -0.38.
